# Lung-gut axis, intestinal microbiota, and pulmonary fibrosis: mechanisms and therapeutic potential

**DOI:** 10.3389/fmicb.2025.1711299

**Published:** 2025-11-28

**Authors:** Jihao Yang, Junwen Wang, Jia Li, Shuo Yang

**Affiliations:** 1 School of Acupuncture and Tuina, Guizhou University of Traditional Chinese Medicine, Guiyang, China; 2 The Second People’s Hospital of Guiyang, Guiyang, China; 3 Guizhou University of Traditional Chinese Medicine Second Affiliated Hospital, Guiyang, China; 4 Guizhou University of Traditional Chinese Medicine Acupuncture Hospital, Guiyang, China

**Keywords:** pulmonary fibrosis, lung-gut axis, intestinal microbiota, microbial mediators, therapeutic potential

## Abstract

Pulmonary fibrosis (PF) is a progressive and life-threatening interstitial lung disease with irreversible lung function loss. The bidirectional interaction between respiratory and gut microbiota mediated by the “lung-gut axis” has emerged as a core regulatory link in PF pathogenesis. This review integrates clinical and preclinical data to systematically clarify the association between microbiota dysbiosis and PF. Clinical evidence shows that PF patients (including idiopathic pulmonary fibrosis, silicosis, and coal workers’ pneumoconiosis) exhibit reduced pulmonary microbiota diversity, increased pro-inflammatory microbial abundance, and altered gut microbiota composition. Preclinical studies using bleomycin or silica-induced PF models confirm consistent microbiota changes and abnormal metabolites. Further, five core pathophysiological mechanisms (immune dysregulation, gut-lung barrier dysfunction, sustained activation of Type 2 epithelial-mesenchymal transition, autophagy modulation, and alveolar epithelial cell apoptosis mediated by microbial peptides) explain how microbiota alterations drive PF progression. Key microbial mediators (e.g., tryptophan metabolites, short-chain fatty acids, lipopolysaccharide, bile acid metabolites) exert bidirectional regulatory effects on PF through synergistic or antagonistic interactions. Additionally, microbiota-targeted strategies such as probiotic/prebiotic intervention, fecal microbiota transplantation, dietary adjustment, and antibiotics have shown experimental anti-fibrotic efficacy. This review highlights the gut microbiota as a potential therapeutic target for PF, while discussing current challenges (e.g., unclear causal relationship, lack of standardized intervention protocols) and future research directions, providing a new framework for PF mechanism research and clinical intervention.

## 1 Introduction

Pulmonary fibrosis (PF) is a progressive and life-threatening interstitial lung disease. It represents the end-stage pathological outcome of various lung disorders, progressing from chronic inflammation to fibrosis. In the end, it causes irreversible loss of lung function and death (Nakamura and Aoshiba, 2016). Its core pathogenesis is driven by the proliferation and differentiation of lung fibroblasts into myofibroblasts. This process is triggered by inflammatory and pro-fibrotic factors secreted after lung injury. It enhances the deposition of extracellular matrix (ECM, mainly composed of collagen fibers) and promotes fibrosis (Wynn, 2011). The lung’s main function is gas exchange, but it is also a key organ that contacts the external environment. During respiration, microorganisms from the outside enter the respiratory tract through the mouth, nose, and pharynx. So, the lower respiratory tract, which was long thought to be sterile, actually has a low-biomass and dynamically changing microbial community (Shukla et al., 2017; Budden et al., 2016; Marsland and Gollwitzer, 2014).

The gut microbiota is a complex community of trillions of bacteria in the gastrointestinal tract. It participates in nutrient absorption, energy supply, and immune regulation in the human body. Thus, it is considered a metabolic organ and a core factor that affects human health and disease (Sonnenburg and Bäckhed, 2016; Muramatsu and Winter, 2024). Many studies have confirmed that intestinal dysbiosis damages the intestinal barrier. Bacterial translocation can worsen immune damage in the lungs (Lin et al., 2018). On the other hand, lung bacteria can change the intestinal microecology through blood circulation and immune responses. At the same time, gut microbiota can affect lung immunity by regulating innate and adaptive immunity (Tan et al., 2020). This bidirectional interaction between the respiratory and gut microbiota is mediated by the circulatory and immune systems. It is defined as the “lung-gut axis” (Wang and Zhang, 2025). It is a core regulatory link in the pathogenesis of PF. Gut microbiota are deeply involved in PF progression via three pathways: cross-organ regulation, cascading reactions induced by respiratory microbiota dysbiosis, and viral co-infection-mediated interference with microbiota-host interactions. It also provides a core theoretical basis for regulating PF through gut microbiota intervention (Luo et al., 2025; Shaheen et al., 2025).

## 2 Evidence for microbiota alterations in PF

**Figure 1:**
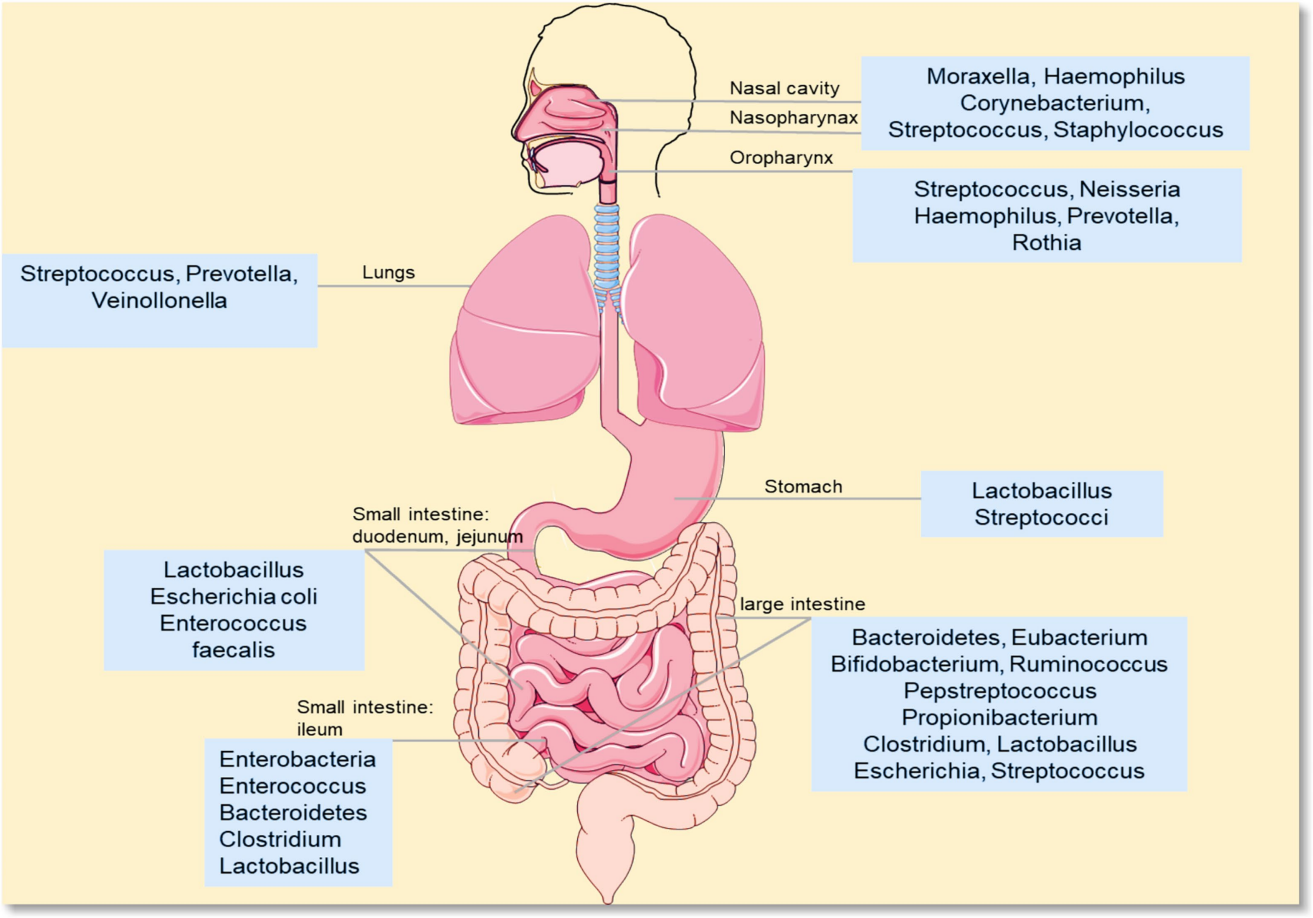
Distribution of different microorganisms. This diagram shows the composition of microbial communities in different parts of the human body (e.g., mouth, gut). Common genera include: oral/upper respiratory tract-related: *Moraxella*, *Haemophilus*, *Streptococcus*, *Neisseria*, *Prevotella*, *Rothia*; gut-related: *Lactobacillus*, *Escherichia coli*, *Enterococcus*, *Bacteroidetes*, *Clostridium*, *Bifidobacterium*; skin/other sites-related: *Staphylococcus*, *Corynebacterium*, *Propionibacterium*.

Based on the theoretical framework of the “lung-gut axis,” the link between microbiota and PF needs support from both clinical patients and animal models. Clinical data can reflect the actual characteristics of microbiota changes in disease states. Animal models can eliminate complex interfering factors and verify the consistency of microbiota changes. This section integrates the characteristics of lung and gut microbiota changes in PF patients (including subtypes such as Idiopathic Pulmonary Fibrosis [IPF], silicosis, and coal workers’ pneumoconiosis [CWP]). It also combines microbiota data from PF animal models induced by bleomycin (BLM) and silica. It clarifies the universality and correlation of microbiota dysbiosis in PF, and provides empirical evidence for subsequent mechanism research (Figure 1).

### 2.1 Clinical evidence of microbiota alterations

Clinical studies have found significant microbiota changes in PF patients. First, there are changes in pulmonary microbiota: In IPF patients, microbial diversity is significantly reduced. Molyneaux et al. (2014) found that IPF patients had a lower Shannon diversity index and fewer bacterial species than healthy people. Takahashi et al. (2018) further confirmed that patients with rapidly progressive IPF had even lower Shannon and Simpson diversity indices. At the same time, the pulmonary microbial load increases and the community composition changes. These are closely related to disease progression and mortality (O’Dwyer et al., 2019; Hérivaux et al., 2022). For example, Han et al. (2014) linked the enrichment of pulmonary Streptococcus to the deterioration of IPF. Patients with silicosis-induced fibrosis show lung-specific microbiota dysbiosis, and the level of lipopolysaccharide (LPS) in bronchoalveolar lavage fluid (BALF) increases (Jia Q. et al., 2024).

Changes in gut microbiota are also obvious in PF patients: Patients with silicosis-induced fibrosis have characteristic dysbiosis. This includes reduced Operational Taxonomic Units (OTUs) and Shannon diversity index, increased abundance of pro-inflammatory microorganisms (e.g., Proteobacteria +47.2%, Verrucomicrobia +32.8%), and decreased beneficial bacteria (e.g., Firmicutes −38.5%) (Ho and Varga, 2017). CWP patients show changes in gut microbiota *β*-diversity. Klebsiella and Haemophilus are positively correlated with the decline in lung function and differential metabolites. This supports an “inflammation–metabolism–fibrosis” axis (Yu et al., 2024).

### 2.2 Animal model evidence of microbiota alterations

Animal models of PF further confirm these microbiota changes. Both pulmonary and gut microbiota show consistent trends. In terms of pulmonary microbiota, BLM-induced PF mice have accelerated and exacerbated fibrosis when exposed to *Streptococcus pneumoniae* and its toxins (Knippenberg et al., 2015). This is consistent with clinical observations of Streptococcus-related IPF deterioration (Han et al., 2014). Silica-induced mice show lung-specific microbiota dysbiosis. It activates the TLR4/NF-κB pathway through increased BALF LPS to drive inflammation-fibrosis (Jia Q. et al., 2024).

In terms of gut microbiota, BLM and silica-induced PF models have 412 differential genera and 26 abnormal metabolites (Zhou et al., 2019). Protective microorganisms (e.g., AlloPrevotella) decrease to 1/7.3 of normal levels, and pathogenic bacteria (e.g., Parasmallomonas) increase to 9.2 times normal (Zhou et al., 2019). Time-dependent changes are also observed. For example, in a rat PF model, the abundance of Lachnospiraceae_NK4A136_group decreases at 2 and 4 weeks. The abundance of Ruminococcaceae_UCG_005 increases temporarily and then decreases significantly (Shaheen et al., 2025). In addition, the top 10 dominant gut microbiota (e.g., Xylobacteraceae, Lactobacillus) show differences between groups. 4 core genera (including Lachnospiraceae_NK4A136_group and Allobaculum) differ between sham-operated, BLM-induced, and silica-induced mice (*p* < 0.05). 8 dominant genera (except Lactobacillus and *Helicobacter pylori*) are more abundant in BLM and silica groups than in the sham-operated group (Gong et al., 2021).

## 3 Core pathophysiological mechanisms linking microbiota to PF

**Figure 2:**
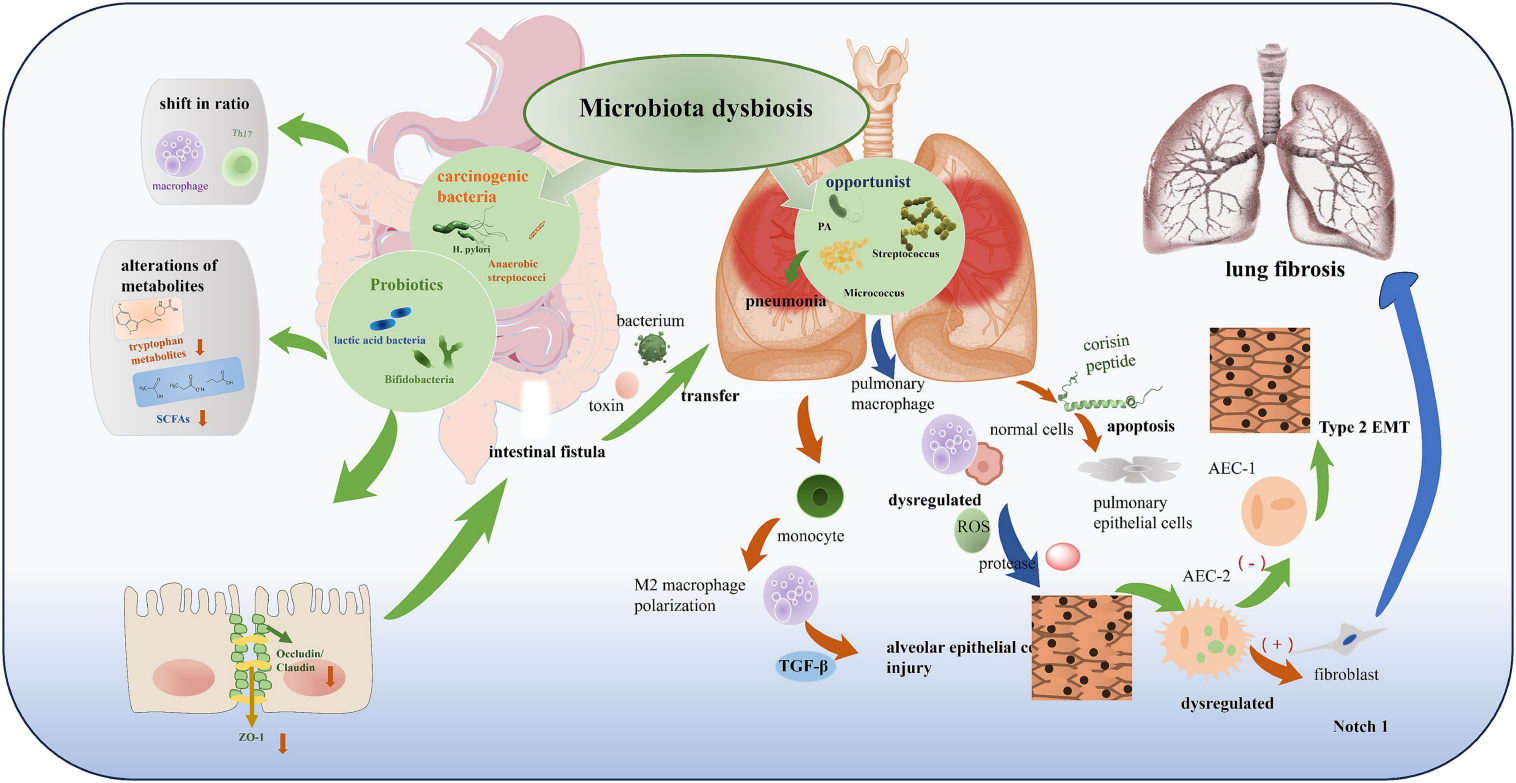
Microbiota dysbiosis in gut-lung axis. According to their effects on the human body, microbiota in dysbiosis can be divided into multiple categories: (1) beneficial (probiotics like lactic acid bacteria, Bifidobacteria); (2) pathogenic/carcinogenic (such as *H. pylori*, anaerobic streptococci); and (3) opportunistic (like *Pseudomonas aeruginosa* (PA), *Streptococcus*, *Micrococcus*). Dysbiosis leads to alterations in microbial composition, such as the proliferation of opportunistic or carcinogenic bacteria, accompanied by alterations of metabolites (e.g., decreased tryptophan metabolites, SCFAs), shift in immune cell ratio (macrophage, Th17), and impairment of intestinal barrier (reduced Occludin/Claudin, ZO-1). This further induces bacterial/toxin transfer, pulmonary inflammation, and ultimately contributes to alveolar epithelial cell injury, Type 2 EMT, and lung fibrosis through pathways like dysregulated ROS, protease, TGF-*β*, and Notch 1.

After clarifying the link between microbiota dysbiosis and PF, the core scientific question turns to “how microbiota drive the progression of PF.” This process is not driven by a single pathway. It is achieved through the coordination of multiple mechanisms such as immune regulation, barrier function, and cell phenotype transformation. Microbiota and their metabolites can gradually promote the transformation of pulmonary inflammation to fibrosis by regulating the balance of immune cells, damaging the integrity of the lung-gut barrier, inducing epithelial-mesenchymal transition (EMT), interfering with autophagy function, and promoting the apoptosis of alveolar epithelial cells. This section analyzes these core pathological mechanisms one by one, and reveals the molecular link between microbiota and PF (Figure 2).

### 3.1 Immune dysregulation

The link between microbiota changes and PF is first mediated by immune dysregulation. Intestinal dysbiosis regulates the activity and function of immune cells to accelerate the progression of PF (Gurczynski et al., 2023; Kletukhina et al., 2022). Microbial metabolites play a key role. Short-chain fatty acids (SCFAs) activate G protein-coupled receptors (GPR43, GPR109A) to balance regulatory T cells (Treg) and Th17 cells (Calvo-Barreiro et al., 2023; Yoon et al., 2023). Tryptophan metabolites regulate T cell differentiation through the aryl hydrocarbon receptor (AhR) pathway (Li J. et al., 2024). In IPF mice, changes in gut microbiota alter the proportions of CD4^+^IL-6^+^ T cells and CD4^+^IL-17A^+^ T cells in lung tissue. It activates the IL-6/STAT3/IL-17A pathway to promote fibrosis (Chioma et al., 2022). Pulmonary microbiota also contribute to inflammation. Bacteroides and Prevotella promote PF through the IL-17R signaling pathway (Yang et al., 2019) and are correlated with IL-17B levels (Parimon et al., 2019). Helicobacter induces lung cells to produce IL-8 and IL-6 (Nakashima et al., 2015). Horizontal transmission of gut microbiota can reduce the severity and mortality of PF by regulating pulmonary CD4^+^IL-10^+^ T cells (Gurczynski et al., 2023).

### 3.2 Barrier dysfunction

Barrier dysfunction further amplifies this pro-fibrotic process. Intestinal dysbiosis directly damages the physical barrier of the intestinal mucosa by downregulating key tight junction proteins (e.g., Occludin, ZO-1, Claudin-1). It triggers “leaky gut” (Wu et al., 2025; He et al., 2025; Hu et al., 2023; Jia M. et al., 2024). This allows intestinal toxins (e.g., LPS) and viable bacteria to translocate into the systemic circulation through the mesenteric lymphatic system or portal circulation, worsening systemic inflammation (Zhao et al., 2025; Qian et al., 2025; Han et al., 2024; Shi et al., 2023). At the same time, dysbiosis reduces SCFA synthesis. SCFA deficiency further impairs the integrity of the intestinal barrier and promotes inflammation (Zheng et al., 2025; Bo et al., 2023; Chen et al., 2021; Sun et al., 2021). These gut-derived endotoxins, bacterial fragments, and inflammatory mediators (e.g., TNF-*α*, IL-6) then reach the lungs through circulation. They damage alveolar epithelial tight junctions and vascular endothelial barriers. For example, LPS activates Toll-like receptors (TLRs) (Saint-Criq et al., 2021) to induce the apoptosis of alveolar epithelial cells, promote the activation of fibroblasts, and accelerate ECM deposition (Keshavarz aziziraftar et al., 2024; Gurczynski et al., 2023; Gong et al., 2021; Ruan et al., 2024). Intestinal dysbiosis also disturbs the pulmonary microbiota (e.g., enrichment of opportunistic pathogens) to disrupt local immune homeostasis (Chen C. et al., 2025). This barrier disruption amplifies the immune-inflammatory cascade: Increased levels of systemic endotoxin and pro-inflammatory factors (e.g., IL-1*β*, IL-17) activate pulmonary macrophages and neutrophils. They release a large amount of reactive oxygen species (ROS) and proteases to damage lung tissue (Li et al., 2021). Reduced gut-derived AhR ligands lead to excessive Th17 responses in the lungs and weakened Treg inhibitory function (Li J. et al., 2024). Changes in bone marrow hematopoiesis promote the recruitment of inflammatory monocytes to the lungs. They differentiate into pro-fibrotic M2-type macrophages that secrete TGF-*β* to stimulate the proliferation of fibroblasts (Gao et al., 2022; Stevens et al., 2022).

### 3.3 EMT

EMT is a key bridge between microbiota dysbiosis and fibrosis. Chronic inflammation induced by microbiota dysbiosis drives the sustained activation of Type 2 EMT. Type 2 EMT is a subtype closely related to tissue repair and fibrosis (Type 1 is for embryonic development, Type 3 is for tumor metastasis) (Kim et al., 2016). Normally, Type 2 EMT stops spontaneously after inflammation subsides. But under the persistent stimulation of microbiota-related inflammation (e.g., activation of the IL-6/STAT3/IL-17A pathway), it persists. This leads to the abnormal accumulation of fibroblasts (Desai et al., 2015). Alveolar epithelial cells (AECs) are critical to this process. Type II AECs (AEC II) are stem cells of the alveolar epithelium. They can self-renew and differentiate into Type I AECs (AEC I) (Chen et al., 2020). But under stress conditions such as inflammation and injury, their normal differentiation into AEC I is inhibited. They initiate EMT to transform into mesenchymal cells. This transformation has become an important “trigger factor” for the formation of PF (Goldmann et al., 2018). The Notch1 signaling pathway regulates the differentiation of AEC II and the progression of fibrosis. Inhibiting Notch signaling improves the differentiation of AEC II and reverses fibrosis (Wasnick et al., 2023).

### 3.4 Autophagy

Autophagy, a process regulated by microbiota, also affects the progression of PF. Microbiota regulate autophagy through multiple pathways. These include secreting metabolites, regulating immune responses, and activating cellular signal cascades (e.g., the PI3K/AKT/mTOR pathway) (Ahangari et al., 2023; Li P. et al., 2023). For example, tetrandrine (Tet) regulates pulmonary microbiota (e.g., Streptococcus, Micrococcus) and their metabolites (e.g., 3,4-dihydroxyphenylpropionic acid, 3,4-DHPPA) to enhance autophagy (Ahangari et al., 2023). Autophagy maintains cellular homeostasis by degrading damaged organelles and proteins. Insufficient autophagy makes alveolar epithelial cells more vulnerable to damage and promotes the progression of fibrosis. Activating autophagy can inhibit the fibrosis process (Bing and Crasta, 2017). Key evidence includes: Inhibiting autophagy by knocking down LC3B and ATG5 induces myofibroblast differentiation (reflected by changes in *α*-smooth muscle actin (α-SMA) and type I collagen expression in lung fibroblasts) (Bing and Crasta, 2017). Autophagy-related protein 7 (ATG7)—an indispensable ubiquitin-activating enzyme in autophagy—induces EMT. Deletion of ATG7 leads to insufficient autophagy and changes in endothelial cell structure. It increases the sensitivity of mice to BLM-induced fibrosis (Singh et al., 2015). Inhibition of autophagy induces EMT through the p62/SQSTM1-NF-κB-Snail2 pathway. It causes local microdamage to senescent alveolar epithelial cells and sustained secretion of pro-fibrotic factors (Hill et al., 2019). TNF-α can downregulate autophagy and increase ROS levels to antagonize TGF-*β*2-induced EMT (Chen et al., 2019).

### 3.5 Alveolar epithelial cell apoptosis

Alveolar epithelial cell apoptosis, mediated by microbiota-derived peptides, further connects microbiota to PF. A study identified a pro-apoptotic peptide called corisin in the pulmonary microbiota. It is a fragment of bacterial transglycosylase present in multiple pathogenic bacterial strains (D’Alessandro-Gabazza et al., 2022). In a mouse model of PF, intratracheal instillation of corisin or bacteria containing corisin led to the apoptosis of alveolar epithelial cells. Clinically, corisin levels were higher in the lungs of slow-progressing IPF patients than in healthy people. Levels in patients with acute exacerbation of PF were significantly higher than in stable PF patients (D’Alessandro-Gabazza et al., 2022). This confirms that alveolar epithelial cell apoptosis is an important transitional mechanism by which microbiota promote PF.

In summary, intestinal and pulmonary microbiota dysbiosis play important roles in the process of pulmonary fibrosis (Table 1). They mainly drive the progression of PF through five coordinated mechanisms. These are immune dysregulation by regulating the balance of immune cells and pathways such as IL-6/STAT3/IL-17A through metabolites, barrier dysfunction that damages the lung-gut barrier to cause “leaky gut” and amplify the inflammatory cascade, epithelial-mesenchymal transition where chronic inflammation leads to abnormal persistence of Type 2 EMT, autophagy regulation that affects autophagy balance through multiple pathways, and alveolar epithelial cell apoptosis mediated by microbiota-derived corisin peptide. At the same time, it points out that these mechanisms have cascade intersections but unclear temporal priority. Key mediators have bidirectional effects, but most studies rely on reversible models that are different from clinically irreversible PF. Due to the low biomass of pulmonary microbiota and the lack of detection standards, the evidence for independent regulatory effects is scattered. These all affect the priority of targeted intervention for PF and clinical transformation.

**Table 1:** Microbiota-related mechanisms in pulmonary fibrosis.

Intervention	Mechanism	Conclusion	References
Qi-Long-Tian capsule	1. Gut microbiota: ↑ α/*β* diversity, ↑ abundance of beneficial bacteria (e.g., Lactobacillus), ↓ pathogenic/pro-inflammatory bacteria; 2. gut-lung axis: Optimizes gut microecology, ↓ intestinal endotoxin translocation, alleviates lung inflammation/fibrosis	Alleviates pulmonary fibrosis via gut microbiota & gut-lung axis	Zhang et al. (2023)
Gut microbiota	1. Structure remodeling: ↑ diversity, regulates SCFA-producing bacteria; 2. Metabolite mediation: SCFAs inhibit pulmonary fibroblast activation/collagen deposition; 3. gut-lung axis: Improves intestinal barrier, ↓ persistence of inflammation after injury	Regulates post-acute lung injury fibrosis in mice via gut-lung axis & SCFAs	Chioma et al. (2022)
Indole-3-acetic acid	1. Lung microbiota: Restores balance, ↑ α diversity, ↓ pro-fibrotic bacteria; 2. Lung microecology: ↓ lung inflammatory factors, indirectly inhibits fibroblast activation	Attenuates pulmonary fibrosis via lung microbiota regulation	Zhuo et al. (2024b)
*Prevotella melaninogenica*	1. Lower respiratory tract: ↑ abundance after checkpoint inhibitors/radiotherapy; 2. Inflammation: Activates local lung immunity, promotes neutrophils/IL-17/TNF-α, exacerbates pneumonia/fibrosis	↑ in lower respiratory tract links to checkpoint inhibitor/radiation pneumonitis	Chen J. et al. (2025)
Silica	1. Lung microbiota: ↓ α diversity, ↑ pro-inflammatory bacteria (e.g., Staphylococcus), ↓ beneficial bacteria; 2. Pathway interaction: Dysbiotic microbiota-derived LPS activates TLR4/NF-κB, triggers inflammation/fibrosis	Lung microbiota dysbiosis contributes to silica-induced fibrosis via LPS/TLR4	Jia Q. et al. (2024)
Phycocyanin	1. Lung microbiota: Restores α diversity/balance of beneficial bacteria (reverses ↓ diversity) in radiation-induced models; 2. Gut microbiota: ↑ beneficial bacteria (e.g., Bifidobacterium), ↓ pro-inflammatory bacteria; 3. gut-lung axis: Alleviates lung radiation injury/fibrosis	Improves microbiota disorder in radiation-induced pulmonary fibrosis via lung-gut microbiota & gut-lung axis	Li et al. (2020)
Gut microbiome-associated metabolites	1. Source: Gut microbiota produces SCFAs, indoles, bile acid derivatives; 2. Mediation: Metabolites regulate macrophage polarization (M1 → M2), inhibit fibroblasts, promote alveolar epithelial repair	Key effectors mediating gut microbiota-regulated pulmonary fibrosis	Li J. et al. (2024)
20(S)-protopanaxadiol	1. Gut microbiota: ↑ SCFA-producing bacteria (e.g., Clostridium), improves intestinal barrier, ↓ endotoxin translocation; 2. gut-lung axis: Inhibits lung inflammation/fibroblast activation	Alleviates pulmonary fibrosis dually via gut microbiota & gut-lung axis	Ruan et al. (2024)
Tetrandrine	1. Lung microbiota metabolism: Alters metabolic profile, ↓ pro-fibrotic metabolites (e.g., lipid metabolites), ↑ anti-fibrotic/anti-inflammatory metabolites; 2. Interaction: Improves alveolar epithelial senescence, alleviates fibrosis	Alleviates pulmonary fibrosis via regulating lung microbiota metabolism	Zhuo et al. (2024a)
Astragalus polysaccharide	1. Gut microbiota: ↑ α diversity in bleomycin models, ↑ beneficial bacteria (Lactobacillus, Bifidobacterium), ↓ pro-inflammatory bacteria; 2. gut-lung axis: Alleviates lung inflammation/fibrosis (plus TLR4/NF-κB inhibition)	Alleviates bleomycin-induced pulmonary fibrosis via gut microbiota & gut-lung axis	Wei et al. (2023)

## 4 Key microbial mediators

The regulation of PF by microbiota does not rely on the direct effect of microbiota itself. It uses their metabolites or bioactive molecules (i.e., “microbial mediators”) as “signal carriers” to transmit regulatory signals between the lung and gut axis, thereby affecting the pathological process of the lungs. These mediators have diverse functions—some can promote inflammation and fibrosis, while others play a protective role. Their balance directly determines the progression direction of PF. This section focuses on tryptophan metabolites, SCFAs, LPS, bile acid metabolites, and other key mediators. It analyzes their bidirectional regulatory roles and molecular targets in PF, and clarifies the “effector molecules” by which microbiota regulate PF.

### 4.1 Tryptophan and its metabolites

In PF, tryptophan and its metabolites have the characteristic of “the parent substance promotes inflammation and fibrosis, while metabolites have bidirectional functions.” As the parent substance, L-tryptophan promotes fibrosis through both *in vitro* and *in vivo* experiments. Under conditions of chronic inflammation and oxidative stress, lung epithelial cells and fibroblasts drive the progression of PF through proliferation, senescence, and EMT (Milara et al., 2018; Kadota et al., 2020). Further differentiation of fibroblasts into myofibroblasts worsens lung tissue damage (Jolly et al., 2017). TGF-*β*1 is a key factor inducing EMT and fibroblast activation (Nakamura and Aoshiba, 2016; Wolters et al., 2014). In BLM- and silica-induced PF mouse models, L-tryptophan treatment further increases the lung/body weight ratio and serum hydroxyproline level. It exacerbates pulmonary inflammatory cell infiltration, alveolar structural damage, and collagen fiber deposition in the ECM. It upregulates the expression of pro-fibrotic marker *α*-SMA and downregulates epithelial cell marker E-cadherin in lung tissue. It also increases the levels of LPS, TNF-α, and IL-1*β* in lung tissue (Nakamura and Aoshiba, 2016).

Tryptophan metabolites show significant functional differences: 5-methoxytryptophan can inhibit the occurrence of PF (Li J. et al., 2024) and alleviate inflammatory responses (Fang et al., 2020). Kynurenine exerts anti-fibrotic activity by antagonizing fibroblast differentiation and promoting collagen degradation (Wang et al., 2016). In contrast, serotonin (5-HT) exacerbates PF by promoting inflammatory responses, protein and cell exudation, and oxidative stress (Dolivo et al., 2018). Notably, indole-3-acetic acid (IAA)—a key tryptophan metabolite—has a clear anti-fibrotic effect. Its levels are significantly reduced in both PF patients and experimental PF mouse models. Its protective mechanisms include three aspects: ① Restoring the autophagic function of TGF-induced fibroblasts by inhibiting the PI3K/AKT/mTOR pathway, thereby reducing fibroblast migration and proliferation; ② Reducing alveolar epithelial cell senescence by regulating the PI3K/AKT and Hif-1 signaling pathways; ③ Altering the composition and structure of the pulmonary microbiota in BLM-induced PF mouse models (Luo et al., 2025). However, existing studies administered IAA 7 days after BLM challenge (simulating clinical treatment scenarios). BLM-induced PF is reversible. Subsequent studies need to verify IAA’s therapeutic effect in more clinically relevant models (e.g., repeated BLM challenge or BLM-challenged senescent mouse models) (Tran et al., 2021).

From the overall perspective of metabolic regulation in the lung-gut axis, the functional balance of tryptophan metabolites relies on a network of multi-component synergy and antagonism: 5-methoxytryptophan (via inhibiting the TGF-*β*/SMAD3 pathway) and kynurenine (via antagonizing fibroblast differentiation) can synergistically enhance anti-fibrotic effects, while both form a clear antagonistic relationship with serotonin (which promotes inflammation and oxidative stress)—this imbalance in their ratios may be a key node in PF progression. Meanwhile, in addition to regulating autophagy on its own, IAA can also synergize with kynurenine to activate the aryl hydrocarbon receptor (AhR) pathway and modulate Treg/Th17 balance. In contrast, the pro-fibrotic effect of exogenous L-tryptophan may indirectly weaken the aforementioned synergistic effects by inhibiting intestinal short-chain fatty acid (SCFA) synthesis, suggesting that subsequent interventions need to consider the coordinated regulation of tryptophan metabolic flux and gut microbiota-derived metabolites.

### 4.2 SCFAs

SCFAs (e.g., acetate, propionate, butyrate) are products of gut microbiota fermentation of dietary fiber. They mediate gut-lung crosstalk to regulate pulmonary immune responses and fibrosis processes. They show clear time-dependent dynamic changes in silicosis models (Zhang et al., 2025). Generally, SCFAs promote the production of regulatory T cells to alleviate pulmonary inflammatory responses (Li X. et al., 2024). They indirectly alleviate pulmonary vascular remodeling by affecting immune cell function in pulmonary hypertension models (a mechanism similar to that in PF) (Yang et al., 2025). They also influence fibrosis development by regulating host metabolic pathways such as amino acid metabolism (Huo et al., 2024). In silicosis model rats, serum acetate levels increase significantly on Day 28, decrease on Days 14/56 (and are lower than in the control group and *Bifidobacterium longum* BB536 intervention group). Propionate concentrations remain stable throughout the process. Butyrate levels tend to increase with disease progression. Total SCFAs peak on Day 28 and then decrease. Total SCFAs in the BB536 group decrease to the lowest level on Day 28 and then increase (Zhang et al., 2025). Further studies confirm that acetate can significantly alleviate alveolar structural damage and collagen deposition in silicosis rats. It also reduces the levels of pulmonary cytokines (IL-1*β*, IL-6, TNF-*α*) and fibrotic markers (type III collagen, α-SMA, vimentin) (Zhang et al., 2025).

From the perspective of metabolite interactions, the SCFA family exhibits characteristics of functional synergy and temporal complementarity: acetate primarily repairs alveolar structure via the Sirt1 pathway, butyrate mainly regulates immune cell polarization, and propionate assists in intestinal barrier repair—these three collectively maintain lung-gut axis homeostasis. More critically, SCFAs form a core antagonistic relationship with lipopolysaccharide (LPS): SCFAs reduce LPS translocation by enhancing the expression of intestinal tight junction proteins (Occludin, ZO-1), while LPS can inhibit the absorption of SCFAs by colonic epithelial cells via the TLR4 pathway. This “mutual restraint” constitutes a core loop for metabolic regulation in the lung-gut axis. Additionally, SCFAs can upregulate the expression of tryptophan hydroxylase in gut microbiota to promote 5-methoxytryptophan production, forming a “SCFA-tryptophan metabolite” synergistic anti-fibrotic axis—its dynamic balance may directly influence the progression of PF.

### 4.3 LPS

As a component of the cell wall of Gram-negative bacteria, LPS is a key pro-inflammatory factor driving PF. It mainly acts through the TLR4/NF-κB pathway. Its sources include local release from pulmonary microbiota dysbiosis and systemic spread from gut microbiota translocation (Jia Q. et al., 2024; Huo et al., 2025). Mechanistically, after LPS activates TLR4 signaling, it can promote alveolar epithelial cell senescence and collagen synthesis (Huo et al., 2025). In a silica-induced PF model, it also activates macrophages and releases inflammatory factors such as IL-6/TNF-α to accelerate ECM deposition (Xu et al., 2025). Experimental evidence shows that antibiotic intervention can reduce LPS levels and alleviate fibrosis (Huo et al., 2025), further verifying the causal association between LPS and PF.

Notably, the pro-fibrotic effect of LPS does not exist in isolation but forms a complex antagonistic network with other key mediators in the lung-gut axis: On one hand, lung-derived LPS activates an inflammatory cascade via the TLR4/NF-κB pathway, while gut microbiota-derived SCFAs can directly antagonize this effect by inhibiting NF-κB phosphorylation. On the other hand, the tryptophan metabolite IAA can reduce the sensitivity of alveolar epithelial cells to LPS-induced apoptosis by repairing autophagic function. Simultaneously, it is necessary to distinguish the synergistic differences between intestinal-derived and lung-derived LPS: Intestinal-derived LPS amplifies systemic inflammation through systemic circulation, while lung-derived LPS directly drives local pulmonary fibrosis. The two may weaken the protective effects of mediators such as SCFAs and IAA through “inflammatory superposition,” suggesting that targeted interventions for LPS need to be differentiated based on their sources.

### 4.4 Bile acid metabolites

Bile acid metabolites are produced by gut microbiota modification of primary bile acids (e.g., lithocholic acid). They exert bidirectional regulatory effects on PF. On one hand, secondary bile acids can bind to the nuclear receptor farnesoid X receptor (FXR) and membrane receptor Takeda G protein-coupled receptor 5 (TGR5) to regulate pulmonary oxidative stress responses and fibroblast activity. The association between bile acid metabolism disorders and disease progression in pulmonary hypertension studies suggests a similar regulatory logic in PF (Huo et al., 2024). On the other hand, bile acid metabolites can synergize with amino acid metabolism (e.g., tryptophan metabolism) to jointly regulate the immune-metabolic network. For example, tryptophan metabolites regulate fibrosis through the AhR (Pan et al., 2025; Yu et al., 2022). Bile acids may participate in this process through signal crosstalk. For instance, cryptotanshinone enriches Enterorhabdus and Akkermansia to activate intestinal bile acid-FXR signaling. It inhibits EMT and pulmonary ECM deposition in radiation-induced PF mice (Li Z. et al., 2023).

Based on the multi-mediator regulatory logic of the lung-gut axis, the anti-fibrotic effect of bile acid metabolites depends on multi-dimensional synergy with other mediators: Secondary bile acids inhibit pulmonary oxidative stress via the FXR/TGR5 pathway, forming functional synergy with the tryptophan metabolite IAA (which repairs alveolar epithelial cells via the PI3K/AKT pathway). Meanwhile, bile acids can promote SCFA synthesis by regulating gut microbiota composition, indirectly enhancing intestinal barrier function to antagonize LPS translocation. Additionally, bile acids can cross-activate with AhR ligands (e.g., kynurenine) to collectively regulate Treg/Th17 immune balance. The synergistic dysregulation of this “bile acid-tryptophan-AhR” axis may be a critical mechanism underlying the dual impairment of intestinal and pulmonary barrier function in PF patients, providing a clear target for subsequent combined targeted interventions.

### 4.5 Other microbial mediators

Other microbial mediators also play regulatory roles in PF: Trimethylamine N-oxide (TMAO)—produced by gut microbiota metabolism of choline—promotes collagen deposition in cardiovascular fibrosis (providing a reference for its role in PF). Traditional Chinese medicine (e.g., Xinshenglong) can reduce TMAO levels and alleviate fibrosis by regulating the gut microbiota (Nie et al., 2025). Urolithin A, a polyphenol secondary metabolite, inhibits AKT1 phosphorylation to block the PI3K/AKT/mTOR pathway. It reduces pulmonary collagen deposition and hydroxyproline while inhibiting fibroblast activation (Ma et al., 2025). 20(S)-protopanaxadiol, a natural product, directly inhibits STING (AMPK/STING-G6PD/SPHK1) and increases SCFA levels by regulating the gut microbiota, thereby alleviating PF (Ruan et al., 2024). From the perspective of a broader mediator interaction network, these non-core metabolites exhibit clear functional complementarity or antagonism with core mediators: The pathological effect of trimethylamine N-oxide (TMAO, which promotes collagen deposition) can be directly antagonized by urolithin A via inhibiting the PI3K/AKT/mTOR pathway, while 20(S)-protopanaxadiol can indirectly weaken TMAO’s pro-fibrotic effect by increasing SCFA levels, forming a closed-loop regulation of “metabolite-microbiota-pathway.” Additionally, urolithin A and IAA exhibit synergy in inhibiting fibroblast activation—the former targets the PI3K/AKT pathway, while the latter regulates autophagy, and their combined use may enhance anti-fibrotic efficacy. Meanwhile, traditional Chinese medicines (e.g., Xinshenglong) can reduce TMAO levels by regulating gut microbiota, suggesting that traditional drugs can exert effects by integrating the microbial mediator network, providing new ideas for multi-target interventions.

In summary, microbiota regulate PF through mediators such as tryptophan metabolites, SCFAs, LPS, bile acid metabolites, TMAO, and urolithin A. The bidirectional functions of mediators and their balance determine the progression of PF (Table 2). However, current studies still face some challenges. The time-dependent dynamic changes of SCFAs in silicosis models (e.g., acetate increases on Day 28 and decreases on Days 14/56) suggest that clinical intervention needs to accurately match the disease course. At the same time, although LPS is clearly a key pro-fibrotic mediator, whether its two sources—pulmonary microbiota dysbiosis and gut microbiota translocation—have differences in intensity or mechanism on the pathological process of PF remains to be further distinguished.

**Table 2:** Gut microbiota metabolites – PF.

Metabolites	Mechanism	Subjects	References
Unsaturated fatty acids, propionic acid, etc. (metabolites after UO₂ exposure)	After uranyl nitrate-induced injury, the intestinal Firmicutes/Bacteroidetes ratio decreases, *Akkermansia* reduces; metabolic pathways show propionic acid metabolism disorder, concurrent with increased TNF-α, IL-1*β*, Col-I, HYP, supporting a “lung injury–intestinal flora–metabolism” positive feedback.	SD rats (inhaled UO₂ aerosol, multiple doses)	Dong et al. (2025)
Urolithin A (a polyphenol secondary metabolite)	Oral UA inhibits AKT1 phosphorylation → blocks PI3K/AKT/mTOR; reduces pulmonary collagen deposition and HYP; *in vitro* verification shows inhibition of fibroblast activation.	C57BL/6 mice (BLM), human pulmonary fibroblasts	Ma et al. (2025)
Multiple lipids/organic acids (UC-MSC-mediated transformation)	UC-MSC modifies intestinal flora to improve α/*β* diversity, while downregulating TNF-α, IL-1*β*, IL-6, TGF-*β*1; speculated to exert anti-fibrosis via the “flora–lipid metabolism–inflammation” pathway.	BLM mice (multiple doses MSC)	Luo et al. (2025)
L-tryptophan	Serum/lung tissue L-Trp is lower in PF mice than in silicosis mice; exogenous L-Trp activates mTOR/S6- to promote EMT and fibroblast activation, aggravating fibrosis.	BLM + silicosis mice; MLE-12 cells, pulmonary fibroblasts	Li J. et al. (2024)
Multiple lipids, amino acids (218 differential metabolites)	Intestinal flora *β*-diversity changes in CWP patients; *Klebsiella*, *Haemophilus* are positively correlated with lung function decline and differential metabolites, suggesting “inflammation–metabolism–fibrosis.”	Humans: 43 cases of coal workers’ pneumoconiosis vs. 48 cases of dust exposure	Yu et al. (2024)
SCFA recovery, G6PD-TCA cycle reprogramming	Natural product 20(S)-PPD directly inhibits STING (AMPK/STING-G6PD/SPHK1) and increases SCFA levels via regulating intestinal flora; vitamins & FMT are essential for flora metabolism.	BLM mice	Ruan et al. (2024)
LPS (lung-derived and gut-related MAMP)	Silicosis causes lung-specific flora dysbiosis → BALF LPS ↑ → activates TLR4-NF-κB, driving inflammation–fibrosis; broad-spectrum antibiotics partially reverse this.	C57BL/6 mice (silicosis)	Jia Q. et al. (2024)
SCFA increase, LPS decrease	Polydatin regulates intestinal flora diversity; FMT (Polydatin→BLM + Abx) can transfer an anti-fibrotic phenotype; inhibits TNF-α, IL-6, IL-1*β*, LPS ↓.	BLM mice; antibiotics + FMT	Yang et al. (2023)
Ursodeoxycholic acid, lithocholic acid, etc. [FXR natural agonists (bile acids)]	Cryptotanshinone enriches *Enterorhabdus*, *Akkermansia* → intestinal bile acid FXR activation; inhibits EMT and pulmonary ECM deposition.	γ-ray induced RILF mice	Li Z. et al. (2023)
SCFA ↑, LPS ↓ (intestinal barrier improver)	Chinese medicine QLT increases *Bacteroidia* / decreases *Clostridia*; improves ZO-1/Claudin/Occludin, sIgA, inhibits TNF-α, IL-6, TGF-*β*1, reduces pulmonary collagen and HYP.	BLM mice (multiple doses)	Yang et al. (2024)

## 5 Microbiota-targeted therapeutic strategies

Based on the core regulatory role of microbiota in PF and the functional characteristics of key mediators, “targeting microbiota to correct dysbiosis” has become a new direction for PF treatment. By intervening in microbiota structure or their metabolites, it is possible to restore the homeostasis of the lung-gut axis and block pro-fibrotic signal transmission. This section sorts out current mainstream microbiota-targeted strategies, including probiotic/prebiotic intervention, Fecal Microbiota Transplantation (FMT), diet and lifestyle adjustment, and antibiotic use. It explains the mechanism of action and experimental efficacy of each strategy (e.g., the downregulating effect of probiotics on inflammation and fibrosis markers, the remodeling effect of FMT on microbiota structure). At the same time, it analyzes the challenges in clinical transformation (e.g., probiotic strain selection, FMT donor standardization), providing new practical ideas for PF treatment.

### 5.1 Probiotic/prebiotic intervention

Probiotic/prebiotic intervention is a widely studied microbiota-targeted strategy for PF. It is based on the link between intestinal dysbiosis (e.g., reduced diversity) and PF. Intestinal dysbiosis can regulate inflammatory responses and immune homeostasis through its metabolites (e.g., SCFAs) to affect pulmonary lesions (Li J. et al., 2024; Zheng et al., 2025). Specific probiotics (e.g., *Bifidobacterium longum* BB536) have shown efficacy: Administering *Bifidobacterium longum* BB536 to silica-induced PF model mice improves pathological damage in lung tissue. It also reduces the levels of pulmonary inflammatory markers (IL-1*β*, IL-6, TNF-*α*) and fibrotic markers (type III collagen, α-SMA, vimentin) (Zhang et al., 2025). Beyond the aforementioned strain, based on existing experimental evidence, two other types of probiotics have demonstrated clear potential in regulating the lung-gut axis to improve PF: *Lactobacillus rhamnosus* GG can inhibit the translocation of intestinal LPS to the lungs by regulating gut microbiota *α*/*β* diversity, while promoting Treg cell proliferation—it significantly alleviates alveolar structural damage in BLM-induced PF models. As an intestinal mucus-degrading bacterium, *Akkermansia muciniphila* shows a decreased abundance closely associated with intestinal barrier damage in PF patients; its supplementation can reduce the infiltration of intestinal-derived inflammatory factors by enhancing the expression of Occludin and ZO-1, and it exhibits synergistic colonization effects with SCFA-producing bacteria. For prebiotics, in addition to traditional dietary fiber, polysaccharides derived from traditional Chinese medicines (e.g., Astragalus polysaccharides, *Lycium barbarum* polysaccharides) can specifically enrich beneficial bacteria such as Lactobacillus and Bifidobacterium, exerting dual effects of microbiota regulation and TLR4/NF-κB pathway inhibition. Based on existing experimental data, a clinical dosage of 10–20 mg/kg/d based on patient weight is recommended, and oral administration combined with nebulization may further improve pulmonary targeting.

Mesenchymal stem cells (MSCs) also exert probiotic-like effects by regulating the gut microbiota: A study using 16S rDNA sequencing found that medium-dose umbilical cord-derived MSCs (UC-MSCs) alter the gut microbiota of IPF mice. At the phylum level, Bacteroidetes decrease and Patescibacteria increase. At the genus level, Prevotellaceae_UCG-001 decreases and Lactobacillus increases. UC-MSCs can inhibit pulmonary inflammatory cell infiltration and fibrosis progression through this regulation (Luo et al., 2025).

Prebiotics, mainly based on dietary fiber, promote the proliferation of beneficial bacteria to increase SCFA production, thereby inhibiting pulmonary inflammation and fibrosis (Ma et al., 2022). Polysaccharide substances (e.g., extracts from traditional Chinese medicine such as Qi-Long-Tian capsule) also exert anti-fibrotic effects by regulating the microbiota. Qi-Long-Tian capsule increases Bacteroidia and decreases Clostridia. It improves the expression of tight junction proteins (ZO-1/Claudin/Occludin) and secretory immunoglobulin A (sIgA). It inhibits the levels of TNF-α, IL-6, and TGF-*β*1, and reduces pulmonary collagen and hydroxyproline (Zhang et al., 2023). However, this intervention method is still in the experimental stage. Questions about dosage, administration methods (e.g., oral colonization), and long-term safety remain unresolved (Song et al., 2023).

### 5.2 FMT

FMT has clear experimental evidence in PF intervention: Transplanting fecal microbiota from healthy mice to PF model mice can reshape the gut microbiota structure (improving α/*β* diversity). It alleviates PF and inflammation (e.g., reducing IL-6, upregulating IL-10). It also inhibits the PF process by regulating immune cells such as CD4^+^IL-10^+^ T cells (Gurczynski et al., 2023). In addition, FMT of microbiota modulated by polydatin can transfer an anti-fibrotic phenotype to BLM-induced mice treated with antibiotics (Yang et al., 2023). At the clinical level, there are currently no data on FMT application in human PF patients. But studies have proposed its potential as a “novel therapeutic strategy.” It also faces challenges such as standardization of donor screening (e.g., clarifying the appropriate microbiota composition and function), optimization of transplantation routes (e.g., selection of colonoscopy/oral capsules), and long-term dynamic monitoring of efficacy (Ruan et al., 2024).

From the standpoint of clinical safety and translational feasibility, the application of FMT in PF patients requires focus on the following core issues: First, infection risk—donor feces may carry latent pathogens such as multi-drug resistant bacteria and Epstein–Barr virus, and PF patients often have impaired immune function, making them susceptible to secondary infections. Based on existing inactivation technologies, a fecal treatment method of heating at 60 °C for 30 min is recommended to reduce this risk. Second, immune rejection—mismatch between donor and recipient gut microecology may trigger local inflammatory responses such as diarrhea and abdominal distension, with an incidence of approximately 15–20%. This can be alleviated by pre-transplant microbiota matching (prioritizing donors with high abundance of SCFA-producing bacteria) and short-term use of low-dose glucocorticoids post-transplant. Third, unknown long-term safety—at least a 2-year follow-up mechanism is required to monitor gut microbiota homeostasis and pulmonary function changes. In terms of clinical operation, the preferred administration route is colonoscopy combined with oral capsules to improve microbiota colonization efficiency; donor screening must include both 16S rDNA sequencing (to exclude pathogenic bacteria) and metabolomic detection (to ensure levels of beneficial metabolites such as SCFAs and IAA). Initially, FMT can be prioritized for patients with acute exacerbation of PF to rapidly inhibit the inflammatory cascade.

### 5.3 Diet and lifestyle intervention

Diet and lifestyle interventions can indirectly affect PF by regulating the gut microbiota: In terms of diet, a high-fiber diet can promote SCFAs production and regulate the gut microbiota-immune axis to inhibit pulmonary inflammation and fibrosis (Gong et al., 2021). A low-fat and low-sugar diet can indirectly reduce the proliferation of harmful bacteria and maintain microbiota homeostasis (though existing literature has not directly associated it with PF) (Li et al., 2022). In lifestyle management, exercise can improve gut microbiota diversity and enhance immunomodulatory function (no direct effect on PF has been found) (Saint-Criq et al., 2021). Smoking cessation can reduce gut microbiota disturbance and pulmonary oxidative damage, potentially delaying fibrosis progression (its specific mechanism still needs verification) (Ma et al., 2022).

### 5.4 Antibiotics

Antibiotics play dual roles in PF intervention: On one hand, they have basic anti-infective and anti-inflammatory capabilities. By inhibiting or killing specific pathogenic microorganisms, they reduce pulmonary inflammation. Some antibiotics have unique anti-fibrotic properties. For example, *α*-azithromycin (a macrolide antibiotic) can promote the apoptosis of primary fibroblasts from IPF patients, exert anti-fibrotic effects, and reduce the expression level of pro-fibrotic genes after TGF stimulation *in vitro* (Krempaska et al., 2020). A 12-month treatment regimen with compound sulfamethoxazole for 181 patients with progressive PF significantly improved patients’ vital capacity and reduced mortality (Shulgina et al., 2012). The current clinical consensus holds that all patients with acute exacerbation of PF should receive broad-spectrum empirical antibiotic treatment even if no clear infection is identified (Maher et al., 2015). Evidence suggests azithromycin is beneficial for acute exacerbation of IPF (Macaluso et al., 2019).

On the other hand, antibiotics are closely associated with microbiota disruption. They can alter the composition of the pulmonary bacterial community (as confirmed in animal models and studies on human pulmonary diseases other than IPF such as chronic obstructive pulmonary disease and lung transplantation) (Dickson et al., 2018; Segal et al., 2016; Rogers et al., 2014; Slater et al., 2013). But it is unclear whether they can change the pulmonary bacterial load—a microbial community characteristic with important prognostic significance in IPF (Invernizzi et al., 2020). For example, long-term azithromycin treatment in patients with chronic obstructive pulmonary disease only changed the microbiota composition, while the total bacterial count remained unchanged (Segal et al., 2016). From the perspective of animal experiments, germ-free mice have significantly reduced mortality in a BLM-induced PF model (O’Dwyer et al., 2019). Pulmonary exposure to *Streptococcus pneumoniae* and its toxins accelerates and exacerbates the fibrosis process (Knippenberg et al., 2015), which is consistent with clinical observations. Multiple antibiotic trials for IPF are ongoing (NCT02759120, 2016), which are expected to provide more evidence for subsequent efficacy evaluation.

**Figure 3:**
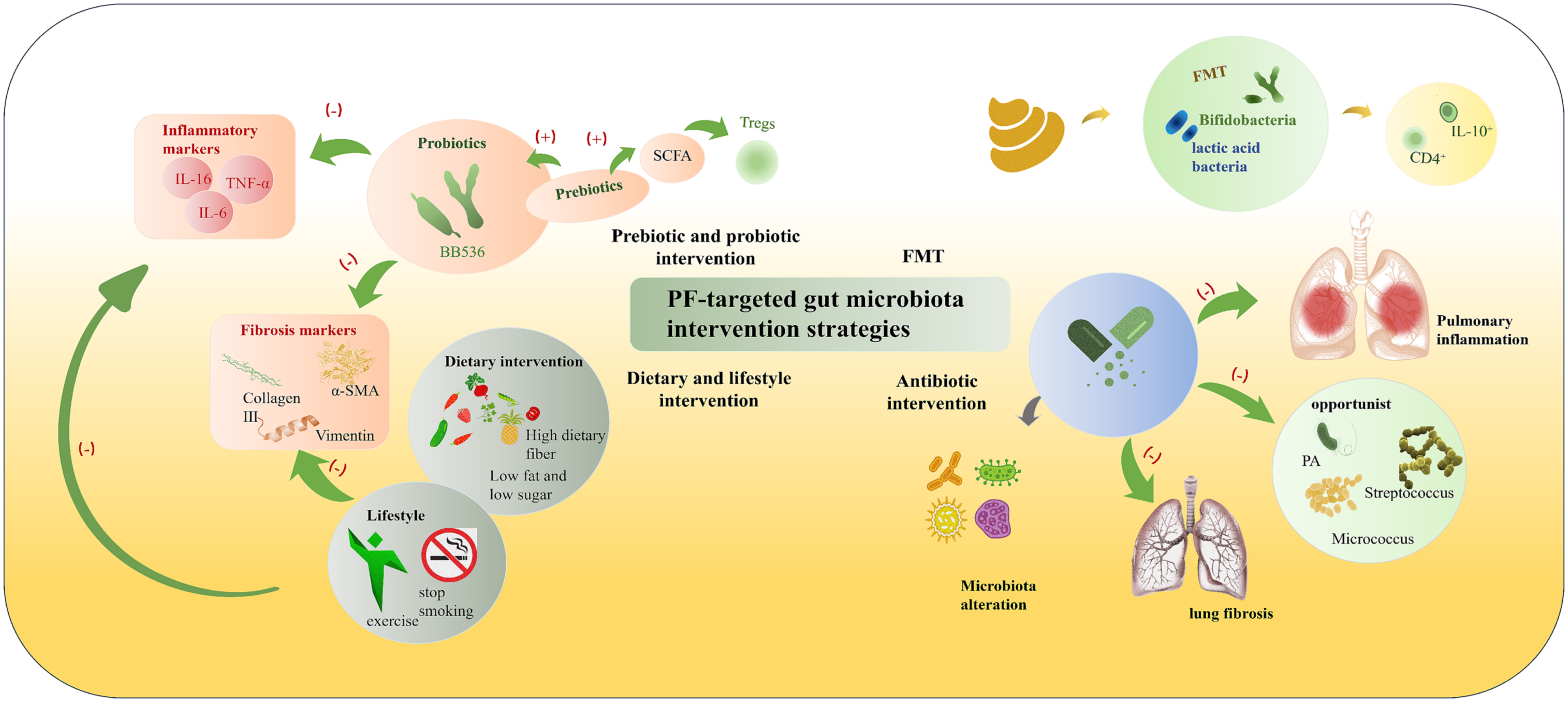
PF-targeted gut microbiota intervention strategies. According to their mechanisms and approaches, interventions for gut microbiota in pulmonary fibrosis can be divided into several categories: (1) Prebiotic and probiotic intervention (probiotics like BB536, prebiotics promoting SCFA production to induce Tregs, thereby inhibiting inflammatory markers such as IL-16, TNF-*α*, IL-6); (2) FMT (Fecal Microbiota Transplantation) (transferring beneficial bacteria like Bifidobacteria, lactic acid bacteria to induce IL-10^+^ CD4^+^ cells); (3) Dietary and lifestyle intervention (high dietary fiber, low fat and low sugar diet, exercise, smoking cessation to reduce fibrosis markers like Collagen III, α-SMA, Vimentin); and (4) Antibiotic intervention (modulating microbiota alteration to inhibit opportunistic bacteria such as PA, Streptococcus, Micrococcus, thereby reducing pulmonary inflammation and lung fibrosis). These strategies collectively target the gut-lung axis to alleviate inflammatory and fibrotic processes.

Microbiota regulation centered on the “lung-gut axis” plays a key role in the pathogenesis of PF. Clinical and animal models confirm the universality of microbiota dysbiosis. Mechanisms such as immune regulation and barrier dysfunction, as well as mediators such as tryptophan metabolites, form a microbiota-PF regulatory network. Strategies such as probiotics and FMT show therapeutic potential. In probiotic/prebiotic intervention, *Bifidobacterium longum* BB536 can improve lung tissue damage in silica-induced PF mice and reduce inflammation and fibrosis markers. Umbilical cord-derived MSCs can inhibit pulmonary fibrosis by regulating the gut microbiota of IPF mice. Prebiotics such as dietary fiber and Qi-Long-Tian capsule can promote beneficial bacteria to produce SCFAs against fibrosis, but there are unresolved issues such as dosage and administration methods (Figure 3). However, FMT can reshape the gut microbiota of PF model mice and alleviate the disease in animal experiments, but there are no data on its application in human PF. It also faces standardization challenges such as donor screening and transplantation routes. Antibiotics (e.g., azithromycin, compound sulfamethoxazole) have both anti-infective and anti-fibrotic effects, but they can damage the pulmonary microbiota. Their impact on lung bacterial load, a key indicator of IPF prognosis, is still unclear. In the future, more attention can be paid to the adaptability from experiments to clinical practice and the possibility of synergy between multiple strategies (e.g., probiotics combined with a high-fiber diet) to improve the accuracy and feasibility of clinical application.

## 6 Conclusion

This work focuses on the pathogenesis of PF. It takes the bidirectional interaction between the respiratory tract and gut microbiota mediated by the “lung-gut axis” as the core. It integrates clinical and preclinical data to clarify the key role of gut microbiota in PF. At the clinical level, IPF patients show reduced respiratory microbiota diversity and increased load. Silicosis and CWP patients exhibit intestinal dysbiosis (reduced beneficial bacteria, increased pro-inflammatory bacteria). At the preclinical level, BLM and silica-induced PF models confirm differential genera (e.g., Lachnospiraceae_NK4A136_group, AlloPrevotella) and abnormal metabolites. Multiple core pathophysiological mechanisms—including immune dysregulation, gut-lung barrier dysfunction, sustained activation of Type 2 EMT, autophagy modulation, and alveolar epithelial cell apoptosis mediated by the bacterial peptide corisin—explain how microbiota alterations drive PF progression. Meanwhile, microbiota-targeted intervention strategies such as probiotics (*Bifidobacterium longum* BB536), FMT, UC-MSCs, high-fiber diet, and antibiotics have shown experimental efficacy. They break the traditional understanding of PF as a “local pulmonary lesion” and provide a new framework for PF mechanism research and clinical intervention. They also confirm that the gut microbiota is a potential new therapeutic target for PF (Song et al., 2023; Ruan et al., 2024).

Current research still faces core challenges: Clinical studies only focus on diagnosed PF patients, making it difficult to clarify the causal relationship between intestinal/respiratory microbiota changes and fibrosis (whether it is a pathogenic factor or a pathological result) (Jia D. et al., 2024). The anti-fibrotic effect of IAA in animal experiments is based on a reversible BLM model, which is different from the irreversible characteristics of clinical PF, limiting the extrapolation of results. At the intervention level, the optimal strain, dosage, and administration method of probiotics, the donor screening, transplantation route, and long-term safety of FMT, and the molecular mechanism of MSCs regulating microbiota have not been clarified. The microbiota-immune-metabolism network regulation is complex. The cross-interaction mechanism between tryptophan metabolites and immune pathways such as IL-6/STAT3/IL-17A has not been clarified (Jia D. et al., 2024; Wang et al., 2024). In clinical transformation, there is a lack of uniform standards for microbial detection and data analysis. The clinical protocols for interventions such as FMT and antibiotics are not standardized. The regulatory effect on lung bacterial load, a key indicator, is unclear (Ma et al., 2022; Gong et al., 2021). Future research needs to make targeted breakthroughs: Conduct longitudinal studies on high-risk populations to clarify the causal relationship between microbiota changes and fibrosis. Optimize animal models to verify the efficacy of key metabolites in irreversible PF. Use multi-omics technology to analyze the cross mechanism of the microbiota-immune-metabolism network (e.g., tryptophan-AhR-IL-17 axis). Establish uniform standards for microbial detection and data analysis and clinical intervention protocols. Explore combined intervention strategies to improve efficacy. Study the regulatory effect of interventions on lung bacterial load and clarify whether it can be used as a therapeutic target (Invernizzi et al., 2020).

Based on the lung-gut axis theory and microbiota-PF regulatory evidence in Lung.docx, two clinically translatable directions deserve focus. For PF patients, specific gut microbiota regulation technologies include targeted probiotic intervention (e.g., *Bifidobacterium longum* BB536 to reduce pulmonary inflammation, *Akkermansia muciniphila* to repair intestinal barriers), optimized FMT (with donor screening via 16S rDNA sequencing and metabolomic detection), and metabolite-strain combination (e.g., IAA plus *Bifidobacterium longum* BB536 for synergistic anti-fibrosis). For PF high-risk populations (e.g., dust-exposed workers), microbiota detection can screen via core indicators: intestinal Firmicutes/Bacteroidetes ratio, pulmonary Streptococcus abundance, and serum LPS/IAA ratio, combined with imaging to enable early warning.

## References

[ref1] Ahangari F. Price N. L. Malik S. Chioccioli M. Bärnthaler T. Adams T. S. . (2023). MicroRNA-33 deficiency in macrophages enhances autophagy, improves mitochondrial homeostasis and protects against lung fibrosis. JCI Insight 8:e158100. doi: 10.1172/jci.insight.158100, PMID: 36626225 PMC9977502

[ref2] Bing C. Crasta K. (2017). “Cellular senescence and autophagy in COPD and IPF” in Autophagy and signaling, 67–90.

[ref3] Bo T. Liu H. Liu M. Liu Q. Li Q. Cong Y. . (2023). Mechanism of inulin in colic and gut microbiota of captive Asian elephant. Microbiome 11:148. doi: 10.1186/s40168-023-01581-3, PMID: 37408039 PMC10324157

[ref4] Budden K. F. Gellatly S. L. Wood D. L. A. Cooper M. A. Morrison M. Hugenholtz P. . (2016). Emerging pathogenic links between microbiota and the gut-lung axis. Nat. Rev. Microbiol. 15, 55–63. doi: 10.1038/nrmicro.2016.142, PMID: 27694885

[ref5] Calvo-Barreiro L. Zhang L. Abdel-Rahman S. A. Naik S. P. Gabr M. (2023). Gut microbial-derived metabolites as immune modulators of T helper 17 and regulatory T cells. Int. J. Mol. Sci. 24:1806. doi: 10.3390/ijms24021806, PMID: 36675320 PMC9867388

[ref6] Chen K. Chen Y. Ueng S. Hwang T. Kuo L. Chen K. J. . (2021). Neutrophil elastase inhibitor (MPH-966) improves intestinal mucosal damage and gut microbiota in a mouse model of 5-fluorouracil-induced intestinal mucositis. Biomed. Pharmacother. 134:111152. doi: 10.1016/j.biopha.2020.111152, PMID: 33373916

[ref7] Chen C. Lin J. Wang X. Yang S. Duan X. Deng Y. . (2025). Novel insights into immune mechanisms in acute lung injury: focusing on gut microbiota and its metabolites. Microbiol. Res. 300:128279. doi: 10.1016/j.micres.2025.128279, PMID: 40695032

[ref8] Chen H. Liu H. Mao M. Tan Y. Mo X. Chen H. T. . (2019). Crosstalk between autophagy and epithelial-mesenchymal transition and its application in cancer therapy. Mol. Cancer 18:101. doi: 10.1186/s12943-019-1030-2, PMID: 31126310 PMC6533683

[ref9] Chen J. Wu H. Yu Y. Tang N. (2020). Pulmonary alveolar regeneration in adult COVID-19 patients. Cell Res. 30, 708–710. doi: 10.1038/s41422-020-0369-7, PMID: 32632255 PMC7338112

[ref10] Chen J. Xu Q. Zhang L. Zhang D. Wu X. (2025). Enrichment of *prevotella melaninogenica* in the lower respiratory tract links to checkpoint inhibitor pneumonitis and radiation pneumonitis. Front. Cell. Infect. Microbiol. 15:1594460. doi: 10.3389/fcimb.2025.1594460, PMID: 41112578 PMC12531214

[ref11] Chioma O. S. Mallott E. K. Chapman A. Van Amburg J. C. Wu H. Shah-Gandhi B. . (2022). Gut microbiota modulates lung fibrosis severity following acute lung injury in mice. Commun. Biol. 5:1401. doi: 10.1038/s42003-022-04357-x, PMID: 36543914 PMC9772329

[ref12] D’Alessandro-Gabazza C. N. Yasuma T. Kobayashi T. Toda M. Abdel-Hamid A. M. Fujimoto H. . (2022). Inhibition of lung microbiota-derived pro-apoptotic peptides ameliorates acute exacerbation of pulmonary fibrosis. Nat. Commun. 13:1558. doi: 10.1038/s41467-022-29064-3, PMID: 35322016 PMC8943153

[ref13] Desai P. Yang J. Tian B. Sun H. Kalita M. Ju H. . (2015). Mixed-effects model of epithelial-mesenchymal transition reveals rewiring of signaling networks. Cell. Signal. 27, 1413–1425. doi: 10.1016/j.cellsig.2015.03.024, PMID: 25862520 PMC4437893

[ref14] Dickson R. P. Erb-Downward J. R. Falkowski N. R. Hunter E. M. Ashley S. L. Huffnagle G. B. (2018). The lung microbiota of healthy mice are highly variable, cluster by environment, and reflect variation in baseline lung innate immunity. Am. J. Respir. Crit. Care Med. 198, 497–508. doi: 10.1164/rccm.201711-2180oc, PMID: 29533677 PMC6118022

[ref15] Dolivo D. M. Larson S. A. Dominko T. (2018). Tryptophan metabolites kynurenine and serotonin regulate fibroblast activation and fibrosis. Cell. Mol. Life Sci. 75, 3663–3681. doi: 10.1007/s00018-018-2880-2, PMID: 30027295 PMC11105268

[ref16] Dong R. Gu X. Su L. Wu Q. Tang Y. Liang H. . (2025). The impact of uranium-induced pulmonary fibrosis on gut microbiota and related metabolites in rats. Meta 15:492. doi: 10.3390/metabo15080492, PMID: 40863111 PMC12388796

[ref17] Fang L. Chen H. Kong R. Que J. (2020). Endogenous tryptophan metabolite 5-methoxytryptophan inhibits pulmonary fibrosis by downregulating TGF-β/SMAD3 and PI3K/AKT signaling. Life Sci. 260:118399. doi: 10.1016/j.lfs.2020.118399 32918977

[ref18] Gao C. Zhou Y. Chen Z. Li H. Xiao Y. Hao W. . (2022). Turmeric-derived nanovesicles as novel nanobiologics for targeted therapy of ulcerative colitis. Theranostics 12, 5596–5614. doi: 10.7150/thno.73650, PMID: 35910802 PMC9330521

[ref19] Goldmann T. Zissel G. Watz H. Drömann D. Reck M. Kugler C. . (2018). Human alveolar epithelial type II cells are capable of TGF-β-dependent epithelial-mesenchymal transition and collagen synthesis. Respir. Res. 19:138. doi: 10.1186/s12931-018-0841-9 30041633 PMC6056940

[ref20] Gong G. Song S. Su J. (2021). Pulmonary fibrosis alters gut microbiota and associated metabolites in mice: an integrated 16S and metabolomics analysis. Life Sci. 264:118616. doi: 10.1016/j.lfs.2020.118616, PMID: 33098825

[ref21] Gurczynski S. J. Lipinski J. H. Strauss J. Alam S. Huffnagle G. B. Ranjan P. . (2023). Horizontal transmission of gut microbiota attenuates mortality in lung fibrosis. JCI Insight 9:e164572. doi: 10.1172/jci.insight.164572, PMID: 38015634 PMC10911107

[ref22] Han M. Wang X. Su L. Pan S. Liu N. Li D. . (2024). Intestinal microbiome dysbiosis increases Mycobacteria pulmonary colonization in mice by regulating the Nos2-associated pathways. Elife 13:RP99282. doi: 10.7554/eLife.99282 39412514 PMC11483126

[ref23] Han M. K. Zhou Y. Murray S. Tayob N. Noth I. Lama V. N. . (2014). Lung microbiome and disease progression in idiopathic pulmonary fibrosis: an analysis of the COMET study. Lancet Respir. Med. 2, 548–556. doi: 10.1016/s2213-2600(14)70069-4, PMID: 24767767 PMC4142525

[ref24] He X. Li M. Zuo X. Ni H. Han Y. He X. D. . (2025). Kushenol I combats ulcerative colitis via intestinal barrier preservation and gut microbiota optimization. World J. Gastroenterol. 31:105656. doi: 10.3748/wjg.v31.i26.105656, PMID: 40678703 PMC12264850

[ref25] Hérivaux A. Willis J. R. Mercier T. Lagrou K. Gonçalves S. M. Gonçales R. A. . (2022). Lung microbiota predict invasive pulmonary aspergillosis and its outcome in immunocompromised patients. Thorax 77, 283–291. doi: 10.1136/thoraxjnl-2020-216179, PMID: 34172558 PMC8867272

[ref26] Hill C. Li J. Liu D. Conforti F. Brereton C. J. Yao L. . (2019). Autophagy inhibition-mediated epithelial-mesenchymal transition augments local myofibroblast differentiation in pulmonary fibrosis. Cell Death Dis. 10:591. doi: 10.1038/s41419-019-1820-x, PMID: 31391462 PMC6685977

[ref27] Ho K. J. Varga J. (2017). Early-life gut dysbiosis: a driver of later-life fibrosis? J. Invest. Dermatol. 137, 2253–2255. doi: 10.1016/j.jid.2017.08.017, PMID: 29055411

[ref28] Hu J. Chen J. Xu X. Hou Q. Ren J. Yan X. (2023). Gut microbiota-derived 3-phenylpropionic acid promotes intestinal epithelial barrier function via AhR signaling. Microbiome 11:102. doi: 10.1186/s40168-023-01551-9, PMID: 37158970 PMC10165798

[ref29] Huo C. Jia Q. Jiao X. Jiang Q. Zeng X. Zhang J. . (2025). Pulmonary microbiota affects silica-induced pulmonary fibrosis through activation of the PI3K/AKT-mediated senescence in alveolar epithelial cells. J. Hazard. Mater. 492:138238. doi: 10.1016/j.jhazmat.2025.138238, PMID: 40233454

[ref30] Huo C. Jiao X. Wang Y. Jiang Q. Ning F. Wang J. . (2024). Silica aggravates pulmonary fibrosis through disrupting lung microbiota and amino acid metabolites. Sci. Total Environ. 945:174028. doi: 10.1016/j.scitotenv.2024.174028, PMID: 38889818

[ref31] Invernizzi R. Barnett J. Rawal B. Nair A. Ghai P. Kingston S. . (2020). Bacterial burden in the lower airways predicts disease progression in idiopathic pulmonary fibrosis and is independent of radiological disease extent. Eur. Respir. J. 55:1901519. doi: 10.1183/13993003.01519-2019, PMID: 31980496 PMC7136009

[ref32] Jia D. Kuang Z. Wang L. (2024). The role of microbial indole metabolites in tumor immunity and therapy. Gut Microbes 16:2409209. doi: 10.1080/19490976.2024.2409209 39353090 PMC11445886

[ref33] Jia M. Liu Y. Liu J. Meng J. Cao J. Miao L. . (2024). Xuanfei Baidu decoction ameliorates bleomycin-elicited idiopathic pulmonary fibrosis in mice by regulating the lung-gut crosstalk via IFN-γ/STAT1/STAT3 axis. Phytomedicine 135:155997. doi: 10.1016/j.phymed.2024.155997, PMID: 39312850

[ref34] Jia Q. Wang H. Wang Y. Xue W. Jiang Q. Wang J. . (2024). Investigation of the mechanism of silica-induced pulmonary fibrosis: the role of lung microbiota dysbiosis and the LPS/TLR4 signaling pathway. Sci. Total Environ. 912:168948. doi: 10.1016/j.scitotenv.2023.168948, PMID: 38048996

[ref35] Jolly M. K. Ward C. Eapen M. S. Myers S. Hallgren O. Levine H. . (2017). Epithelial–mesenchymal transition, a spectrum of states: role in lung development, homeostasis and disease. Dev. Dyn. 247, 346–358. doi: 10.1002/dvdy.24541, PMID: 28646553

[ref36] Kadota T. Yoshioka Y. Fujita Y. Araya J. Minagawa S. Hara H. . (2020). Extracellular vesicles from fibroblasts induce epithelial-cell senescence in pulmonary fibrosis. Am. J. Respir. Cell Mol. Biol. 63, 623–636. doi: 10.1165/rcmb.2020-0002oc, PMID: 32730709

[ref37] Keshavarz aziziraftar S. Bahrami R. Hashemi D. Shahryari A. Ramezani A. Ashrafian F. . (2024). The beneficial effects of *Akkermansia muciniphila* and its derivatives on pulmonary fibrosis. Biomed. Pharmacother. 180:117571. doi: 10.1016/j.biopha.2024.117571, PMID: 39418965

[ref39] Kim K. K. Sisson T. H. Horowitz J. C. (2016). Fibroblast growth factors and pulmonary fibrosis: it's more complex than it sounds. J. Pathol. 241, 6–9. doi: 10.1002/path.4825, PMID: 27757968 PMC5499705

[ref40] Kletukhina S. Mutallapova G. Titova A. Gomzikova M. (2022). Role of mesenchymal stem cells and extracellular vesicles in idiopathic pulmonary fibrosis. Int. J. Mol. Sci. 23:11212. doi: 10.3390/ijms231911212, PMID: 36232511 PMC9569825

[ref41] Knippenberg S. Ueberberg B. Maus R. Bohling J. Ding N. Tort Tarres M. . (2015). *Streptococcus pneumoniae* triggers progression of pulmonary fibrosis through pneumolysin. Thorax 70, 636–646. doi: 10.1136/thoraxjnl-2014-206420, PMID: 25964315 PMC6729139

[ref42] Krempaska K. Barnowski S. Gavini J. Hobi N. Ebener S. Simillion C. . (2020). Correction to: azithromycin has enhanced effects on lung fibroblasts from idiopathic pulmonary fibrosis (IPF) patients compared to controls. Respir. Res. 21:29. doi: 10.1186/s12931-020-1304-7, PMID: 31992294 PMC6986083

[ref43] Li Z. Dong J. Wang M. Yan J. Hu Y. Liu Y. . (2022). Resveratrol ameliorates liver fibrosis induced by nonpathogenic Staphylococcus in BALB/c mice through inhibiting its growth. Mol. Med. 28:52. doi: 10.1186/s10020-022-00463-y, PMID: 35508992 PMC9066969

[ref44] Li P. Hao X. Liu J. Zhang Q. Liang Z. Li X. . (2023). miR-29a-3p regulates autophagy by targeting Akt3-mediated mTOR in SiO2-induced lung fibrosis. Int. J. Mol. Sci. 24:11440. doi: 10.3390/ijms241411440, PMID: 37511199 PMC10380316

[ref45] Li W. Lu L. Liu B. Qin S. (2020). Effects of phycocyanin on pulmonary and gut microbiota in a radiation-induced pulmonary fibrosis model. Biomed. Pharmacother. 132:110826. doi: 10.1016/j.biopha.2020.110826, PMID: 33068929 PMC7556228

[ref46] Li X. Shang S. Wu M. Song Q. Chen D. (2024). Gut microbial metabolites in lung cancer development and immunotherapy: novel insights into gut-lung axis. Cancer Lett. 598:217096. doi: 10.1016/j.canlet.2024.217096, PMID: 38969161

[ref47] Li Z. Shen Y. Xin J. Xu X. Ding Q. Chen W. . (2023). Cryptotanshinone alleviates radiation-induced lung fibrosis via modulation of gut microbiota and bile acid metabolism. Phytother. Res. 37, 4557–4571. doi: 10.1002/ptr.7926, PMID: 37427974

[ref48] Li J. Wu W. Kong X. Yang X. Li K. Jiang Z. . (2024). Roles of gut microbiome-associated metabolites in pulmonary fibrosis by integrated analysis. NPJ Biofilms Microbiomes 10:154. doi: 10.1038/s41522-024-00631-4, PMID: 39702426 PMC11659409

[ref49] Li S. Zhuge A. Wang K. Lv L. Bian X. Yang L. . (2021). Ketogenic diet aggravates colitis, impairs intestinal barrier and alters gut microbiota and metabolism in DSS-induced mice. Food Funct. 12, 10210–10225. doi: 10.1039/d1fo02288a, PMID: 34542110

[ref50] Lin Z. Ye W. Zu X. Xie H. Li H. Li Y. . (2018). Integrative metabolic and microbial profiling on patients with spleen-yang-deficiency syndrome. Sci. Rep. 8:6619. doi: 10.1038/s41598-018-24130-7, PMID: 29700349 PMC5920061

[ref51] Luo Y. Zhou S. Zhang X. Lin Y. Liu J. Cheng W. . (2025). The role of the microbiota and metabolites in the treatment of pulmonary fibrosis with UC-MSCs: integrating fecal metabolomics and 16S rDNA analysis. PLoS One 20:e0313989. doi: 10.1371/journal.pone.0313989, PMID: 39787138 PMC11717254

[ref52] Ma J. Wang W. Gao K. Dong Z. (2025). Urolithin a attenuates pulmonary fibrosis via the PI3K/AKT/mTOR pathway: evidence from network pharmacology and experimental validation. Biochem. Biophys. Res. Commun. 776:152219. doi: 10.1016/j.bbrc.2025.152219, PMID: 40554052

[ref53] Ma P. Wang M. Wang Y. (2022). Gut microbiota: a new insight into lung diseases. Biomed. Pharmacother. 155:113810. doi: 10.1016/j.biopha.2022.113810, PMID: 36271581

[ref54] Macaluso C. Maritano Furcada J. Alzaher O. Chaube R. Chua F. Wells A. U. . (2019). The potential impact of azithromycin in idiopathic pulmonary fibrosis: a hypothesis. Eur. Respir. J. 53:1800628. doi: 10.1183/13993003.00628-2018 30442715

[ref55] Maher T. M. Whyte M. K. B. Hoyles R. K. Parfrey H. Ochiai Y. Mathieson N. . (2015). Development of a consensus statement for the definition, diagnosis, and treatment of acute exacerbations of idiopathic pulmonary fibrosis using the Delphi technique. Adv. Ther. 32, 929–943. doi: 10.1007/s12325-015-0249-6, PMID: 26498943 PMC4635174

[ref56] Marsland B. J. Gollwitzer E. S. (2014). Host–microorganism interactions in lung diseases. Nat. Rev. Immunol. 14, 827–835. doi: 10.1038/nri3769, PMID: 25421702

[ref57] Milara J. Hernandez G. Ballester B. Morell A. Roger I. Montero P. . (2018). The JAK2 pathway is activated in idiopathic pulmonary fibrosis. Respir. Res. 19:24. doi: 10.1186/s12931-018-0728-9, PMID: 29409529 PMC5801676

[ref58] Molyneaux P. L. Cox M. J. Willis-Owen S. A. G. Mallia P. Russell K. E. Russell A. M. . (2014). The role of bacteria in the pathogenesis and progression of idiopathic pulmonary fibrosis. Am. J. Respir. Crit. Care Med. 190, 906–913. doi: 10.1164/rccm.201403-0541oc, PMID: 25184687 PMC4299577

[ref59] Muramatsu M. K. Winter S. E. (2024). Nutrient acquisition strategies by gut microbes. Cell Host Microbe 32, 863–874. doi: 10.1016/j.chom.2024.05.011, PMID: 38870902 PMC11178278

[ref60] Nakamura H. Aoshiba K. (2016). Idiopathic pulmonary fibrosis. Japan: Springer.

[ref61] Nakashima S. Kakugawa T. Yura H. Tomonaga M. Harada T. Hara A. . (2015). Identification of *Helicobacter pylori* VacA in human lung and its effects on lung cells. Biochem. Biophys. Res. Commun. 460, 721–726. doi: 10.1016/j.bbrc.2015.03.096, PMID: 25817795 PMC6118350

[ref38] NCT02759120. (2016). CleanUP IPF for the Pulmonary Trials Cooperative. Available online at: https://clinicaltrials.gov/show/nct02759120

[ref62] Nie Q. Zhao J. Haseeb S. Deng S. Zhang X. Wang R. . (2025). Xinshuaining preparation ameliorates doxorubicin-induced cardiac injury in heart failure rats by regulating gut microbiota. Drug Deliv. Transl. Res. doi: 10.1007/s13346-025-01879-9, PMID: 40419735

[ref64] O’Dwyer D. N. Ashley S. L. Gurczynski S. J. Xia M. Wilke C. Falkowski N. R. . (2019). Lung microbiota contribute to pulmonary inflammation and disease progression in pulmonary fibrosis. Am. J. Respir. Crit. Care Med. 199, 1127–1138. doi: 10.1164/rccm.201809-1650oc, PMID: 30789747 PMC6515865

[ref65] Pan Y. Deng Y. Yang H. Yu M. (2025). The aryl hydrocarbon receptor: a promising target for intestinal fibrosis therapy. Pharmacol. Res. 219:107909. doi: 10.1016/j.phrs.2025.107909, PMID: 40812693

[ref66] Parimon T. Yao C. Habiel D. M. Ge L. Bora S. A. Brauer R. . (2019). Disturbed lung flora promotes pulmonary fibrosis by regulating interleukin-17B production via secretory epithelial vesicles. JCI Insight 4:e129359. doi: 10.1172/jci.insight.129359 PMC6777916 31393853

[ref67] Qian S Su Z Lin J Hou Q Wang X Li Y 2025. Inhibition of Farnesoid-X-receptor signaling during abdominal sepsis by dysbiosis exacerbates gut barrier dysfunction. Cell Commun Signal. 23:236. doi: 10.1186/s12964-025-02224-w 40399878 PMC12096737

[ref68] Rogers G. B. Bruce K. D. Martin M. L. Burr L. D. Serisier D. J. (2014). The effect of long-term macrolide treatment on respiratory microbiota composition in non-cystic fibrosis bronchiectasis: an analysis from the randomised, double-blind, placebo-controlled BLESS trial. Lancet Respir. Med. 2, 988–996. doi: 10.1016/s2213-2600(14)70213-9, PMID: 25458200

[ref69] Ruan Y. Ren G. Wang M. Lv W. Shimizu K. Zhang C. (2024). The dual role of 20(S)-protopanaxadiol in alleviating pulmonary fibrosis through the gut-lung axis. Phytomedicine 129:155699. doi: 10.1016/j.phymed.2024.155699, PMID: 38733907

[ref70] Saint-Criq V. Lugo-Villarino G. Thomas M. (2021). Dysbiosis, malnutrition and enhanced gut-lung axis contribute to age-related respiratory diseases. Ageing Res. Rev. 66:101235. doi: 10.1016/j.arr.2020.101235, PMID: 33321253

[ref71] Segal L. N. Clemente J. C. Wu B. G. Wikoff W. R. Gao Z. Li Y. . (2016). Randomised, double-blind, placebo-controlled trial with azithromycin selects for anti-inflammatory microbial metabolites in the emphysematous lung. Thorax 72, 13–22. doi: 10.1136/thoraxjnl-2016-208599, PMID: 27486204 PMC5329050

[ref72] Shaheen N. Miao J. Xia B. Zhao Y. Zhao J. (2025). Multifaceted role of microbiota-derived indole-3-acetic acid in human diseases and its potential clinical application. FASEB J. 39:e70574. doi: 10.1096/fj.202500295r, PMID: 40415505 PMC12104694

[ref73] Shi L. Jin L. Huang W. (2023). Bile acids, intestinal barrier dysfunction, and related diseases. Cells 12:1888. doi: 10.3390/cells12141888, PMID: 37508557 PMC10377837

[ref74] Shukla S. D. Budden K. F. Neal R. Hansbro P. M. (2017). Microbiome effects on immunity, health and disease in the lung. Clin. Transl. Immunol. 6:e133. doi: 10.1038/cti.2017.6, PMID: 28435675 PMC5382435

[ref75] Shulgina L. Cahn A. P. Chilvers E. R. Parfrey H. Clark A. B. Wilson E. C. . (2012). Treating idiopathic pulmonary fibrosis with the addition of co-trimoxazole: a randomised controlled trial. Thorax 68, 155–162. doi: 10.1136/thoraxjnl-2012-202403, PMID: 23143842

[ref76] Singh K. K. Lovren F. Pan Y. Quan A. Ramadan A. Matkar P. N. . (2015). The essential autophagy gene ATG7 modulates organ fibrosis via regulation of endothelial-to-mesenchymal transition. J. Biol. Chem. 290, 2547–2559. doi: 10.1074/jbc.m114.604603, PMID: 25527499 PMC4317030

[ref77] Slater M. Rivett D. W. Williams L. Martin M. Harrison T. Sayers I. . (2013). The impact of azithromycin therapy on the airway microbiota in asthma. Thorax 69, 673–674. doi: 10.1136/thoraxjnl-2013-204517, PMID: 24287164 PMC4078717

[ref78] Song W. Yue Y. Zhang Q. (2023). Imbalance of gut microbiota is involved in the development of chronic obstructive pulmonary disease: a review. Biomed. Pharmacother. 165:115150. doi: 10.1016/j.biopha.2023.115150, PMID: 37429232

[ref79] Sonnenburg J. L. Bäckhed F. (2016). Diet–microbiota interactions as moderators of human metabolism. Nature 535, 56–64. doi: 10.1038/nature18846, PMID: 27383980 PMC5991619

[ref80] Stevens J. Steinmeyer S. Bonfield M. Peterson L. Wang T. Gray J. . (2022). The balance between protective and pathogenic immune responses to pneumonia in the neonatal lung is enforced by gut microbiota. Sci. Transl. Med. 14:eabl3981. doi: 10.1126/scitranslmed.abl3981, PMID: 35704600 PMC10032669

[ref81] Sun Y. He Z. Li J. Gong S. Yuan S. Li T. . (2021). Gentamicin induced microbiome adaptations associate with increased BCAA levels and enhance severity of influenza infection. Front. Immunol. 11:608895. doi: 10.3389/fimmu.2020.608895, PMID: 33708192 PMC7940682

[ref82] Takahashi Y. Saito A. Chiba H. Kuronuma K. Ikeda K. Kobayashi T. . (2018). Impaired diversity of the lung microbiome predicts progression of idiopathic pulmonary fibrosis. Respir. Res. 19:34. doi: 10.1186/s12931-018-0736-9, PMID: 29486761 PMC6389110

[ref83] Tan J. Tang Y. Huang J. (2020). Gut microbiota and lung injury. Adv. Exp. Med. Biol. 1238, 55–72. doi: 10.1007/978-981-15-2385-4_5, PMID: 32323180

[ref84] Tran T. Assayag D. Ernst P. Suissa S. (2021). Effectiveness of proton pump inhibitors in idiopathic pulmonary fibrosis: a population-based cohort study. Chest 159, 673–682. doi: 10.1016/j.chest.2020.08.2080, PMID: 32882251

[ref85] Wang G. Fan Y. Zhang G. Cai S. Ma Y. Yang L. . (2024). Microbiota-derived indoles alleviate intestinal inflammation and modulate microbiome by microbial cross-feeding. Microbiome 12:59. doi: 10.1186/s40168-024-01750-y, PMID: 38504383 PMC10949743

[ref86] Wang Y. Hsu Y. Wu H. Lee G. Yang Y. Wu J. Y. . (2016). Endothelium-derived 5-methoxytryptophan is a circulating anti-inflammatory molecule that blocks systemic inflammation. Circ. Res. 119, 222–236. doi: 10.1161/circresaha.116.308559, PMID: 27151398

[ref87] Wang Y. Zhang Y. (2025). Research progress on the correlation of gut microbiota with pulmonary tuberculosis based on the theory of exterior and interior of lung and large intestine. Chin. Med. Cult. 8, 40–49. doi: 10.1097/mc9.0000000000000127

[ref88] Wasnick R. Korfei M. Piskulak K. Henneke I. Wilhelm J. Mahavadi P. . (2023). Notch1 induces defective epithelial surfactant processing and pulmonary fibrosis. Am. J. Respir. Crit. Care Med. 207, 283–299. doi: 10.1164/rccm.202105-1284oc, PMID: 36047984

[ref89] Wei Y. Qi M. Liu C. Li L. (2023). *Astragalus* polysaccharide attenuates bleomycin-induced pulmonary fibrosis by inhibiting TLR4/NF-κB signaling pathway and regulating gut microbiota. Eur. J. Pharmacol. 944:175594. doi: 10.1016/j.ejphar.2023.175594, PMID: 36804541

[ref63] Wolters P. J. Collard H. R. Jones K. D. (2014). Pathogenesis of idiopathic pulmonary fibrosis. Annu Rev Pathol. 9, 157–179. doi: 10.1146/annurev-pathol-012513-104706 24050627 PMC4116429

[ref90] Wu S. Gao S. Lin D. Bekhit A. E. A. Chen Y. (2025). Intestinal barrier restoration in UC: dietary protein/peptide mediates microbiota-Trp-AhR axis and food processing implications. Food Res. Int. 217:116799. doi: 10.1016/j.foodres.2025.116799, PMID: 40597515

[ref91] Wynn T. A. (2011). Integrating mechanisms of pulmonary fibrosis. J. Exp. Med. 208, 1339–1350. doi: 10.1084/jem.20110551, PMID: 21727191 PMC3136685

[ref92] Xu C. Sun P. Jiang Q. Meng Y. Dong L. Wang X. . (2025). Tissue-resident *Klebsiella quasipneumoniae* contributes to progression of idiopathic pulmonary fibrosis by triggering macrophage mitophagy in mice. Cell Death Discov. 11:168. doi: 10.1038/s41420-025-02444-6, PMID: 40221415 PMC11993561

[ref93] Yang D. Chen X. Wang J. Lou Q. Lou Y. Li L. . (2019). Dysregulated lung commensal bacteria drive interleukin-17B production to promote pulmonary fibrosis through their outer membrane vesicles. Immunity 50, 692–706.e7. doi: 10.1016/j.immuni.2019.02.001, PMID: 30824326

[ref94] Yang C. Du Y. Li Q. Liu L. Zhao L. Gao C. . (2024). Fructo-oligosaccharides alleviated ulcerative colitis via gut microbiota-dependent tryptophan metabolism in association with aryl hydrocarbon receptor activation in mice. J. Agric. Food Chem. 72, 27912–27922. doi: 10.1021/acs.jafc.4c07248, PMID: 39641614

[ref95] Yang J. Liu J. Gu H. Song W. Zhang H. Wang J. . (2025). Gut microbiota, metabolites, and pulmonary hypertension: mutual regulation and potential therapies. Microbiol. Res. 299:128245. doi: 10.1016/j.micres.2025.128245, PMID: 40480048

[ref96] Yang J. Shi X. Gao R. Fan L. Chen R. Cao Y. . (2023). Polydatin alleviates bleomycin-induced pulmonary fibrosis and alters the gut microbiota in a mouse model. J. Cell. Mol. Med. 27, 3717–3728. doi: 10.1111/jcmm.17937, PMID: 37665061 PMC10718135

[ref97] Yoon J. Do J. Velankanni P. Lee C. Kwon H. (2023). Gut microbial metabolites on host immune responses in health and disease. Immune Netw. 23:e6. doi: 10.4110/in.2023.23.e6, PMID: 36911800 PMC9995988

[ref98] Yu H. Feng Z. Lin W. Yang K. Liu R. Yu H.-X. . (2022). Ongoing clinical trials in aging-related tissue fibrosis and new findings related to AhR pathways. Aging Dis. 13:732. doi: 10.14336/ad.2021.1105, PMID: 35656117 PMC9116921

[ref99] Yu X. Xiong T. Yu L. Liu G. Yang F. Li X. . (2024). Gut microbiome and metabolome profiling in coal workers' pneumoconiosis: potential links to pulmonary function. Microbiol. Spectr. 12:e0004924. doi: 10.1128/spectrum.00049-24, PMID: 39283109 PMC11537036

[ref100] Zhang Q. Luo T. Yuan D. Liu J. Fu Y. Yuan J. (2023). Qi-long-Tian capsule alleviates pulmonary fibrosis development by modulating inflammatory response and gut microbiota. Funct. Integr. Genomics 23:64. doi: 10.1007/s10142-023-00988-3, PMID: 36810971

[ref101] Zhang W. Qi X. Han M. Jia Q. Li X. Yin W. . (2025). Activation of Sirt1 by acetate alleviates silicofibrosis: contribution of the gut microbiota. Ecotoxicol. Environ. Saf. 292:117969. doi: 10.1016/j.ecoenv.2025.117969, PMID: 40020386

[ref102] Zhao H. Lin G. Yin Y. Wu Q. Wang Y. Tang N. . (2025). Impact of micro- and nanoplastics on gastrointestinal diseases: recent advances. Eur. J. Intern. Med. 139:106419. doi: 10.1016/j.ejim.2025.07.015, PMID: 40701881

[ref103] Zheng L. Fu Y. Wu J. Liu T. Zhang X. Qu C. . (2025). Modulation of gut microbiota by traditional Chinese medicine: a novel therapeutic approach for chronic inflammatory airway diseases. Am. J. Chin. Med. 53, 2043–2070. doi: 10.1142/s0192415x25500764, PMID: 40947646

[ref104] Zhou Y. Chen L. Sun G. Li Y. Huang R. (2019). Alterations in the gut microbiota of patients with silica-induced pulmonary fibrosis. J. Occup. Med. Toxicol. 14:5. doi: 10.1186/s12995-019-0225-1, PMID: 30867671 PMC6399897

[ref105] Zhuo J. Chu L. Liu D. Zhang J. Chen W. Huang H. . (2024a). Tetrandrine alleviates pulmonary fibrosis by modulating lung microbiota-derived metabolism and ameliorating alveolar epithelial cell senescence. Phytother. Res. 39, 298–314. doi: 10.1002/ptr.8374, PMID: 39510818

[ref106] Zhuo J. Liu D. Yu Q. Hu M. Huang H. Chen Y. . (2024b). Indole-3-acetic acid attenuates pulmonary fibrosis by modulating lung microbiota, inhibiting fibroblast activation, and alleviating alveolar epithelial cell senescence. Life Sci. 359:123191. doi: 10.1016/j.lfs.2024.123191, PMID: 39481838

